# Dantrolene versus amiodarone for cardiopulmonary resuscitation: a randomized, double-blinded experimental study

**DOI:** 10.1038/srep40875

**Published:** 2017-01-18

**Authors:** Thomas Wiesmann, Dennik Freitag, Wolfgang Dersch, Daphne Eschbach, Marc Irqsusi, Thorsten Steinfeldt, Hinnerk Wulf, Carsten Feldmann

**Affiliations:** 1 Department of Anesthesia and Intensive Care, University Hospital Marburg, Baldingerstrasse, 35033 Marburg, Germany; 2 Department of Orthopedic and Trauma Surgery, University Hospital Marburg, Baldingerstrasse, 35033 Marburg, Germany; 3 Department of Cardiothoracic Surgery, University Hospital Marburg, Baldingerstrasse, 35033 Marburg, Germany

## Abstract

Dantrolene was introduced for treatment of malignant hyperthermia. It also has antiarrhythmic properties and may thus be an alternative to amiodarone for the treatment of ventricular fibrillation (VF). Aim of this study was to compare the return of spontaneous circulation (ROSC) with dantrolene and amiodarone in a pig model of cardiac arrest. VF was induced in anesthetized pigs. After 8 min of untreated VF, chest compressions and ventilation were started and one of the drugs (amiodarone 5 mg kg^−1^, dantrolene 2.5 mg kg^−1^ or saline) was applied. After 4 min of initial CPR, defibrillation was attempted. ROSC rates, hemodynamics and cerebral perfusion measurements were measured. Initial ROSC rates were 7 of 14 animals in the dantrolene group vs. 5 of 14 for amiodarone, and 3 of 10 for saline). ROSC persisted for the 120 min follow-up in 6 animals in the dantrolene group, 4 after amiodarone and 2 in the saline group (n.s.). Hemodynamics were comparable in both dantrolene group amiodarone group after obtaining ROSC. Dantrolene and amiodarone had similar outcomes in our model of prolonged cardiac arrest, However, hemodynamic stability was not significantly improved using dantrolene. Dantrolene might be an alternative drug for resuscitation and should be further investigated.

Amiodarone is the drug of choice for refractory ventricular fibrillation in cardiopulmonary resuscitation (CPR) after a third unsuccessful attempt at defibrillation. Dantrolene, which effects calcium regulation and is used to reduce metabolic rate in malignant hyperthermia, has potent antiarrhythmic properties; it was superior to saline in an unblinded experimental CPR study in pigs^1^.

Ventricular fibrillation (VF) results in increased diastolic calcium leaks from the sarcoplasmatic reticulum via ryanodine receptor subtype 2 (RyR2) within cardiac myocytes. Dantrolene decreases calcium leakage by RyR2 and increases the threshold for spontaneous calcium release as well resulting in improved RyR2 function without alteration of contractile function^1^,^2^. Published experimental data in small animal studies showed the potential of dantrolene to mediate normalization of VF induced dysfunctional calcium cycling resulting in more successful defibrillation attempts and decreased refibrillation rates^1^.

However, in animals in which the return of spontaneous circulation (ROSC) was obtained with dantrolene, resulting hemodynamics were poorly investigated. The aim of this study was to compare dantrolene with amiodarone regarding ROSC rates in an experimental model of prolonged ventricular fibrillation (8 min) in pigs. Additionally, hemodynamics as well as cerebral perfusion and oximetry were noted in surviving animals.

## Methods

### Study design and ethical approval

This study was carried out in accordance with the national animal protection laws; the protocol was approved by local authorities (RP Giessen, V 54–19 c 2015 h 01 MR 20/13 Nr.20/2015). It was performed on 38 anesthetized pigs (Sus scrofa domestica, German Land Race, age 10–14 weeks) in an established experimental model for cardiopulmonary resuscitation^3^,^4^.

### Anesthesia and instrumentation

**Figure 1:**
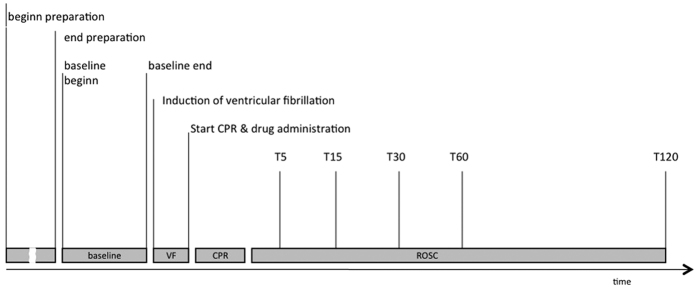
Flow Chart–Experimental setup.

In brief, animals were premedicated using ketamine 20 mg kg^−1^, atropine 0.03 mg kg^−1^ and diazepam 1 mg kg^−1^ for intramuscular use. After obtaining peripheral venous access via an ear vein, anesthesia was induced with sufentanil (1 μg kg^−1^) and propofol (3–6 mg kg^−1^). Endotracheal intubation was performed with an ID 5.5–6.0 mm endotracheal tube. Maintenance of anesthesia was with sufentanil (1–2 μg kg^−1^ h) and propofol (2–3 mg kg^−1^ h). Animals were then placed supine and vascular access was performed using ultrasound-guided catheter insertion. Right jugular veins (for pulmonary artery catheter placements) as well as left femoral veins were used for cannulation. A right femoral arterial line was inserted for blood sampling (at baseline, 0 (T0), 5 (T5), 15 (T15), 30 (T30), 60 (T60) and 120 (T120) minutes post ROSC, see Fig. 1) and measuring arterial blood pressure. Pulse oximetry, capnography as well as a 3 lead ECG were also used.

**Figure 2:**
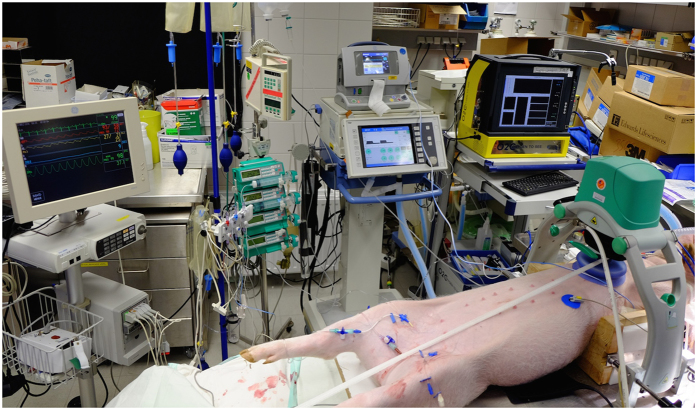
O2c probe application.

To achieve a standardized chest compression model, animals were placed supine in an adjustable U-shaped frame for optimal application of a pneumatic driven chest compression device (LUCAS 1, 100 compressions per minute, compression only mode, Physio-Control, Neuss, Germany). External defibrillation pads were attached on both sides of the thorax and connected to a biphasic defibrillator (Responder 2000, GE Healthcare, Freiburg, Germany). An overview of the experimental setting is given in Fig. 2.

**Figure 3:**
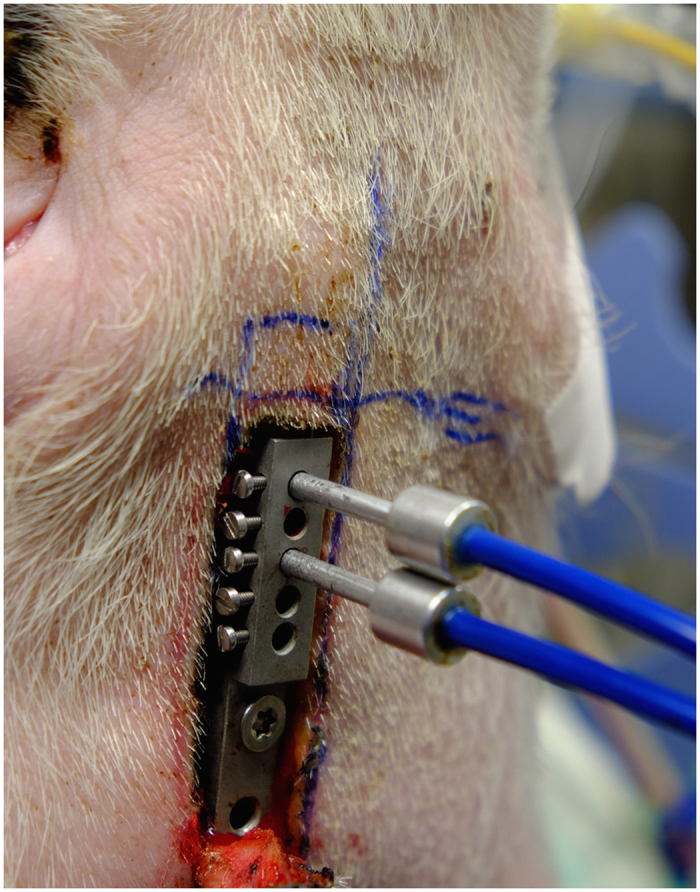
Experimental setup–Overview.

To measure cerebral forebrain oxygenation and perfusion, two holes were drilled in the frontal bone in a standardized manner as described before^3^. Cerebral measurements of nerve blood flow (flow in AU, arbitrary units) and cerebral oxygen saturation (ScO2%) were obtained using Laser spectrophotometry (O2C, LEA Medizintechnik, Giessen, Germany) after insertion of the respective probes. The system and probe were calibrated for accuracy according to the manufacturer’s recommendation. The probe was applied to the exposed dura mater with a custom-made application device (Fig. 3).

The O2C machine consists of two different units. First, forebrain blood flow (flow) is measured by laser Doppler flowmetry. Second, the tissue spectrophotometry unit calculates tissue hemoglobin content and oximetry. In brief, the underlying tissue is illuminated with coherent laser light of 500–630 nm wavelength and 30-mW power through a fiberoptic cable. Probe geometry allowed a detection of blood flow by analyzing backscattered light. The ScO2 of the capillary and postcapillary microvasculature is calculated by the O2c device, by fitting measured spectra with spectra of known tissue saturation values (ScO2). Cerebral oximetry (ScO2) is given as percentage, flow values were measured as arbitrary units and calculated as percentage changes to baseline values.

During a steady state period before the start of the experiments, animals were ventilated using an inspiratory oxygen fraction of 0,25 to achieve an arterial PaCO2 of 40 mmHg using a tidal volume of 6–8 ml kg^−1^ (Evita, Dräger Medical, Lübeck, Germany).

### Experimental protocol

Ventricular fibrillation (VF) was induced with an AC 7.5–15 V current via a paced electrode in the right ventricle. After induction of VF, animals were disconnected from the ventilator and left untreated for 8 minutes without any ventilation, chest compression or other intervention. After this, a standardized resuscitation protocol was started. Mechanical chest compressions were performed using the LUCAS device with a fixed rate of 100/min. Standardized ventilation was initiated at an FiO2 of 1,0 with volume-controlled intermittent positive pressure ventilation (PEEP 5mbar, Vt 8 ml kg^−1^, respiratory rate 12/min, I:E 1:1.5). At the start of resuscitation, one of the drugs (amiodarone 5 mg kg^−1^, dantrolene 2.5 mg kg^−1^ or saline (sham) was applied in a blinded fashion via the indwelling femoral venous catheter. The same infused fluid volumes were used for the interventional drugs. After 4 min of the initial standardized chest compressions and ventilation (to simulate a basic life support (BLS) sequence), defibrillation with 200 J was attempted, and was repeated after each CPR cycle according to current guidelines^4^,^5^.

In the event of prolonged VF, VT without ROSC or another episode of VT or VF after achieved ROSC, the respective study drug was given again in half of the initial dosage (amiodarone 2.5 mg kg^−1^ (Cordarex, Sanofi Aventis, Frankfurt, Germany, solved in 5% glucose solution according to the manufacturer’s recommendation), dantrolene 2.5 mg kg^−1^ (Dantrolen IV, Norgine, Marburg, Germany, solved in water for injection as delivered by the manufacturer) or isotonic saline infusion (NaCl, B. Braun, Melsungen, Germany) according to current CPR guidelines^5^,^6^. If ROSC was not achieved after four defibrillation cycles, no further CPR attempts were made. No epinephrine or other drugs were given during the experiments. If spontaneous circulation returned, chest compressions were commenced for 1 min. Afterwards, cardiovascular status was monitored but no drugs given to modulate cardiac or vascular status. If cardiac arrest recurred, standardized cardiopulmonary resuscitation as per protocol was applied as described above for up to 5 CPR cycles until successful ROSC or study termination.

Secondary VF or pulseless VT occurring after primary ROSC was treated with half the initial dosage of the study drug if this had not been already given. Ventilator settings were adjusted according to blood gas analysis performed at predetermined intervals.

The study was terminated on the death of the respective animal, or, if ROSC was successful and survival lasted 120 min or more (T120), the animals were euthanized with a lethal dose of potassium chloride.

### Randomization & Blinding

To obtain blinding, the infusion bottle and IV line were completely masked by an opaque coating. Randomization to the study groups (using sealed envelopes) and drug preparation was performed by a research assistant who was not responsible for any part of the resuscitation or post-ROSC treatment. Study investigators were unaware of the randomization results until study completion and data analysis.

### Study endpoints

The primary endpoints were firstly, ROSC (defined as maintenance of systolic BP ≥ 60 mmHg for a minimum of 5 minutes) and secondly, sustained ROSC, defined as persistent spontaneous circulation until 120 min after successful defibrillation^7^.

Secondary endpoints were hemodynamic variables such as cardiac output, heart rate, arterial blood pressures, ECG patterns (QRS widths, QT times), blood gas analysis and cerebral oxygenation and perfusion measurements. Analysis and group comparisons were to be performed hierarchically, firstly by comparisons between the dantrolene and amiodarone groups. Statistical analysis of the saline group regarding secondary outcome parameters were to be performed only if at least 4 animals achieved spontaneous circulation at each post-ROSC time point.

### Sample Size Calculation

Sample size calculation was based on published data for ROSC rates for amiodarone and dantrolene in previous experimental studies. Assuming a ROSC difference (persistent ROSC at 120 min) of 45% between amiodarone and dantrolene^1^,^8^, an alpha of 0.05 and a beta of 0.80, a sample size of n = 14 per group was calculated using G* Power (G* Power, Release 3.1, F. Faul *et al*., Heinrich Heine University, Düsseldorf, Germany)^2^,^9^. For the saline control group we decided to use ten animals to minimize animal numbers according to ethical reasons.

### Statistics

Data are presented as median with 25^th^–75^th^ percentiles (IQR, interquartile range) for non-normally distributed parameters or as numbers (n) and frequencies (%) for qualitative parameters. Group comparisons were performed between dantrolene and amiodarone groups using Fisher’s exact test or Mann-Whitney tests. In the event of a p-value of <0.05, pairwise comparisons were made using a hierarchical test approach. First, dantrolene and amiodarone groups were compared; secondly, comparisons between dantrolene or amiodarone and the saline group were performed. Additional comparisons with the saline group were to be made only, if sufficient data was obtained for a given time point (n > 4). Statistical analysis was performed using SPSS (release 22, SPSS IBM, Chicago, IL, USA).

## Results

38 animals (mean weight 41.7 kg) were anesthetized and randomized to one of three groups (dantrolene, n = 14; amiodarone, n = 14; saline, n = 10). There were no significant differences in hemodynamic or other variables between groups at baseline, and no differences in ROSC rates between dantrolene and amiodarone (7/14 vs. 5/14, not significant). At 120 min of follow up, ROSC rates were comparable in the intervention groups (6/14 vs. 4/14, not significant). ROSC rates were not different when the dantrolene and amiodarone were compared with saline. Similar numbers of shocks were needed to achieve initial successful ROSC between groups (Table 1). Animals in the amiodarone group received 210 mg (195–220) of the study drug amiodarone (median, 25^th^–75^th^ percentiles), whereas the dantrolene group animals received 105 mg (92.5–107.5) dantrolene. Repeated study drug bolus according to protocol was given in one animal of the dantrolene group with ROSC until T120 min, whereas none of the surviving amiodarone group animals received a second bolus of the study drug. Of the saline group, only two animals had an sustained ROSC until T120 min. According to predefined criteria, secondary parameters were compared only between dantrolene and amiodarone. Baseline hemodynamics (before induction of cardiac arrest) were comparable between all three group. In the intervention groups, cardiac output was not significantly greater in the dantrolene group for each post-ROSC time point (T5 min–T120 min). Heart rate was not significantly different between dantrolene and amiodarone group. Post-ROSC, mean arterial pressure as well as systolic and diastolic blood pressure were comparable in the dantrolene and the amiodarone group. Mean arterial pressure (MAP) was similar post-ROSC (Table 2). Only at T5 min did the dantrolene group have a significantly higher systolic blood pressure (150 vs. 95 mmHg, p = 0.045).

Mean and diastolic blood pressures did not differ between groups for given time points. Blood gas analysis showed comparable values regarding pH, paCO2 and BE (base excess) and lactate (Table 3). Regarding cerebral perfusion and oxygenation, measurements showed similar non-significant changes in both interventional groups during time (all p-values > 0.05, Table 4).

## Discussion

Our proof-of concept study showed no advantage of dantrolene over amiodarone regarding the primary outcomes of ROSC and sustained ROSC during a 120 min follow-up. Additionally, cardiac output as well as arterial blood pressure data of animals with ROSC showed no significant difference between both study groups.

Amiodarone reduces blood pressure, and is associated with negative inotropy and bradycardia^6^,^10^. Current ERC guidelines recommend it for refractory VF or pulseless VT (i.e. after three shocks)^3^,^5^. The optimal time for application (early versus late) during CPR is not known. There are only a few studies of early application for amiodarone, which show equivocal results^5^,^7^,^8^,^11^,^12^,^13^.

A recently published randomized, double-blinded study comparing amiodarone or lidocaine with placebo for out-of-hospital arrest showed no improved overall or favorable neurological outcome for both drugs^14^. This study challenges the role of amiodarone for treatment of cardiac arrest and stresses the need for new therapeutic options in cardiac arrest. We assume, that amiodarone will no longer be the standard antiarrhythmic drug in future recommendations due to the deleterious effects on hemodynamics as well as the lack of improving outcome after cardiac arrest compared with placebo. This might be explainable by the parenteral formulation of amiodarone containing polysorbate as discussed below. The well-documented beneficial effects of enteral application of amiodarone for chronic therapy of ventricular arrhythmias remains undisturbed in this context.

The recent pig study of Karlis *et al*. compares early administration of amiodarone, combined with epinephrine, with saline in a model of prolonged ventricular fibrillation. Despite the use of epinephrine during CPR, diastolic aortic and coronary perfusion pressures were significantly lower immediately after ROSC with amiodarone. Overall ROSC rates were similar^8^. Only one study compared dantrolene with saline in a model of cardiac arrest with 4 minutes of ventricular fibrillation. In the dantrolene group, Zamiri *et al*. showed superior hemodynamics and earlier termination of ventricular fibrillation^1^, but there was neither a comparison with amiodarone nor an adequate blinding of the respective study drug. Overall survival rates were similar to those of saline. Thus, we performed this randomized and double-blinded study of prolonged (8 minutes) ventricular fibrillation to achieve greater insight into early post-ROSC hemodynamics, cerebral perfusion and cerebral oximetry (ScO2) in a robust and established model. The lack of significantly different ROSC rates between groups might be attributable to the small number of study animals per group. Contrary to our ROSC results, Zamiri showed better ROSC rates at the end of their study period (dantrolene 85% vs. saline 39%) at 30 min after start of the resuscitation. Compared with our study, our ROSC rates were 43% (dantrolene), 29% (amiodarone) and 20% (saline) at the time point 2 h after start of the resuscitation. These differences might be explainable by the longer time period of untreated cardiac arrest (8 min vs. 4 min. in the study by Zamiri) and a longer time between start of basic life support and first defibrillation attempt (4 min vs. 3 min) in our study, which is more realistic regarding clinical practice and might have resulted in more treatment-resistent cardiac arrest. However, a sample size calculation was done to perform the study with sufficient animal numbers regarding this primary endpoint. Our sample size corresponds to the sample size in the study (n = 13 per group) by Zamiri *et al*.^1^ Amongst secondary outcomes, the dantrolene group had no statistically significant improved blood pressure or cardiac output, important for organ perfusion. However, this result should be interpreted carefully as the numbers of animals with ROSC were not high enough to achieve an appropriate sample size for robust statistic evaluation. Further trials should be powered for sufficient numbers of surviving animals (with ROSC) for appropriate statistical analysis. Nevertheless, our hemodynamic data might be helpful for others to perform sample size calculations for own projects. In the dantrolene group, heart rate for each given time point was not statistically different from the amiodarone group. Regarding arterial blood gas analysis, there were no differences between groups. Dantrolene is known to induce a slight hypokalemia, which might be of importance in a post-ROSC setting. Interestingly, in our study, potassium levels were comparable between both interventional groups.

Dantrolene is poorly soluble; a conventional adult dose requires dissolving 8–10 bottles of dantrolene powder in a solvent. This would limit its use in the CPR setting^15^. The recent introduction of azumolene, a dantrolene analogue which is 30 times more water-soluble^16^ may present an feasible alternative for CPR. Further pharmacological developments in the future might result in more specific RyR2-modulating drugs with acceptable handling properties (solubility) and better pharmacological effects than azumolene or dantrolene.

Ours was an animal study using a well-established study design and transfer of its results to clinical situations should be made with caution, especially because we used young animals without pre-existing cardiac pathology or cardiovascular medication. Parenteral amiodarone solutions regularly contain polysorbate 80, which may result in relevant mean arterial blood pressure reductions^17^. A polysorbate-free amiodarone solution is marketed in the US since 2008 but not available in most other countries worldwide^18^. Further studies should evaluate this polysorbate-free amiodarone solution in context of cardiopulmonary resuscitation to delineate the effects of amiodarone on ROSC rates and hemodynamics without the potential detrimental effects of the component polysorbate 80 on hemodynamics.

A further limitation of the present study in this context is that we studied the effects of dantrolene and amiodarone in anaesthetized animals, while resuscitation in clinical practice is predominantly done in unconscious patients who are neither sedated nor anaesthetized. The pigs were anaesthetized with propofol, known to affect heart rate, inotropy and vascular tone. This might be expected to interfere with the hemodynamic reaction to amiodarone or dantrolene^19^. However, studying CPR outcome in pig models is regularly performed with anesthetized animals due to ethical reasons. Further studies should evaluate a high-soluble dantrolene solution (azumolene) in comparison with a standard group with polysorbate-free amiodarone and a third group without any antiarrhythmic drug. To approximate a clinical scenario, further studies should be performed using epinephrine according to current international guidelines. These studies must be appropriately powered for the evaluation of hemodynamic outcome data in animals with ROSC.

## Conclusions

In this experimental blinded study of prolonged ventricular fibrillation, ROSC rates were not significantly different with early administration of dantrolene and amiodarone. Secondary outcome parameters like hemodynamics were not significantly different between study groups but should be interpreted cautiously due to small numbers of animals with ROSC. Further experimental studies should evaluate the effects of dantrolene in combination with a guideline-adhered resuscitation protocol, which includes epinephrine.

## Additional Information

**How to cite this article:** Wiesmann, T. *et al*. Dantrolene versus amiodarone for cardiopulmonary resuscitation: a randomized, double-blinded experimental study. *Sci. Rep.* **7**, 40875; doi: 10.1038/srep40875 (2017).

**Publisher's note:** Springer Nature remains neutral with regard to jurisdictional claims in published maps and institutional affiliations.

## Figures and Tables

**Table 1:** ROSC rates.

	Dantrolene (n = 14)	Amiodarone (n = 14)	Saline (n = 10)
ROSC ever	7	5	3
ROSC persistent	6	4	2
Shocks until ROSC	1 (1–3)	2 (2–3)	1 (1–4)

Data are presented as absolut numbers (%) or median (25^th^–75^th^ percentiles). No. significant comparisons were found between groups using Fisher’s Exact Test, level of significance p < 0.05.

**Table 2:** Hemodynamics.

	Baseline		T5		T15		T30		T60		T120	
	Dantrolene	Amiodarone	Dantrolene	Amiodarone	Dantrolene	Amiodarone	Dantrolene	Amiodarone	Dantrolene	Amiodarone	Dantrolene	Amiodarone
HR (min-1)	93 (88–119)	94 (78–111)	156 (153–214)	159 (145–163)	151 (116–179)	132 (109–134)	145 (138–169)	126 (113–140)	145 (130–160)	117 (112–128)	131 (127–136)	94 (86–102)
MAP (mmHg)	79 (71–85)	87 (77–93)	100 (71–135)	61 (53–84)	67 (53–131)	62 (30–64)	86 (75–84)	58 (55–64)	75 (65–84)	65 (51–75)	85 (77-87)	70 (63–75)
SAP (mmHg)	100 (94–112)	108 (98–115)	150 (132–173)	95 (67–121)*	121 (85–171)	100 (50–103)	110 (90–128)	66 (64–75)	93 (78–98)	80 (62–80)	107 (95–110)	89 (85–94)
DAP (mmHg)	64 (59–70)	73 (62–84)	76 (47–110)	44 (41–64)	49 (41–90)	27 (19–44)	68 (58–77)	50 (47–53)	60 (54–65)	54 (49–59)	72 (66–74)	59 (52–64)
CO (l/min)	4.7 (3.8–5.3)	4.0 (3.6–4.8)	6.05 (4.9–6.5)	3.4 (1.9–4.2)	4.4 (4.3–5.3)	4.0 (2.0–4.4)	4.3 (3.4–4.7)	2.5 (2.3–2.6)	3.65 (3.5–3.8)	2.6 (2.45–2.70)	3.6 (2.9–3.8)	3.05 (2.75–3.35)

Data are expressed as median (25^th^–75^th^ percentiles). HR, heart rate. MAP, mean arterial pressure. SAP, systolic arterial pressure. DAP, diastolic arterial pressure. CO, cardiac output. T5-T120, time points 5 min until 120 min after initial ROSC. *Significant, Level of significance p < 0.05. Bonferroni adjustments were made for accounting statistical testing of multiple time points. For details see text.

**Table 3:** Blood gas analysis.

	Baseline		T5		T15		T30		T60		T120	
	Dantrolene	Amiodarone	Dantrolene	Amiodarone	Dantrolene	Amiodarone	Dantrolene	Amiodarone	Dantrolene	Amiodarone	Dantrolene	Amiodarone
pH	7.48 (7.45–7.50)	7.45 (7.42–7.47)	7.18 (7.14–7.27)	7.18 (7.15–7.19)	7.19 (7.17–7.24)	7.23 (7.11–7.26)	7.25 (7.21–7.32)	7.28 (7.23–7.79)	7.33 (7.28–7.36)	7.34 (7.3–7.39)	7.38 (7.35–7.43)	7.38 (7.34–7.44)
pCO2 (mmHg)	40 (38–42)	41 (41–43)	50 (46–69)	59 (53–67)	57 (53–64)	53 (46–64)	51 (48–60)	48 (43–55)	49 (47–56)	43 (36–52)	47 (45–52)	43 (37–49)
BE (mmol/l)	6 (4.2–7.4)	4.4 (3.3**–**6.1)	−5.4 (−9.2–−5.0)	−7 (−7.1–−6.0)	−5.2 (−6.5–−3.5)	−6 (−7.7–−5.4)	−3.8 (−4.1–−1.7)	−4.4 (−5.9–−4.3)	−0.2 (−0.9–1.1)	−1.3 (−3.3–−0.8)	3.3 (2-5–4.2)	1.9 (−0.6–2.8)
pO2 (mmHg)	96 (90–104)	88 (83–100)	482 (241–551)	494 (490–518)	496 (473–541)	521 (508–528)	526 (494–547)	525 (508–562)	541 (487–553)	554 (543–575)	555 (532–568)	571 (568–573)
Sodium (mmol/l)	138 (137–139)	137 (136–139)	133 (128–134)	137 (134–137)	136 (133–138)	138 (136–138)	136 (133–137)	137 (135–137)	136 (133–136)	137 (137–137)	134 (134–135)	138 (137–138)
Potassium (mmol/l)	4.0 (3.8–4.1)	3.9 (3.7–4.1)	5.5 (4.5–6.1)	4.7 (4.7–5.0)	3.7 (3.4–3.9)	3.3 (3.2–3.3)	4.1(3.8–4.1)	3.6 (3.3–3.8)	4.6 (4.4–4.7)	3.9 (3.8–3.9)	5.6 (5.1–5.7)	4.2 (4.1–4.5)
Lactate (mmol/l)	1.4 (0.9–1.8)	1.3 (0.8–2.2)	7.2 (6.2–10.2)	8.6 (7.7–8.8)	7.4 (6.3–8.8)	7.4 (6.5–8.7)	6.2 (5.0–7.4)	6.6 (6–7.6)	4.1 (3.5–5.2)	5.0 (4.3–5.5)	1.9 (1.7–2.6)	2.5 (2.2–3.6)
Glucose (mg/dl)	110 (100–121)	98 (94–109)	228 (123–308)	295 (287–312)	175 (136–314)	273 (248–359)	166 (154–270)	237 (225–338)	178 (123–215)	205 (162–250)	139 (119–160)	153 (114–194)

Data are expressed as median (25^th^–75^th^ percentiles). PaO2, arterial oxygen tension; PaCO2, arterial carbon dioxide tension. BE, base excess. No significant differences were shown comparing both groups for variables at certain time points. Level of significance p < 0.05. Bonferroni adjustments were made to compensate for multiple testing.

**Table 4:** Brain oximetry and perfusion measurements.

	Baseline		T5		T15		T30		T60		T120	
	Dantrolene	Amiodarone	Dantrolene	Amiodarone	Dantrolene	Amiodarone	Dantrolene	Amiodarone	Dantrolene	Amiodarone	Dantrolene	Amiodarone
ScO2 (AU)	37 (28–41)	37 (31–39)	65 (53–82)	74 (35–76)	65 (52–74)	51 (46–73)	46 (31–49)	38 (29–50)	42 (30–46)	36 (27–54)	41 (31–70)	35 (29–45)
Flow (AU)	85 (75–121)	72 (63–111)	100 (62–185)	60 (59–108)	107 (60–156)	73 (54–91)	88 (66–105)	68 (59–85)	86 (65–133)	74 (63–99)	80 (64–122)	77 (63–99)
